# Is there an optimal sequence of biologic therapies for inflammatory bowel disease?

**DOI:** 10.1177/17562848231159452

**Published:** 2023-04-05

**Authors:** Brian Bressler

**Affiliations:** The IBD Centre of BC, University of British Columbia, 770-1190 Hornby Street, Vancouver, BC V6Z 2K5, Canada

**Keywords:** biologic therapy, Crohn’s disease, inflammatory bowel disease, ulcerative colitis

## Abstract

Over the past two decades, 11 biologic agents have been approved for use in most countries for the treatment of moderate-to-severe inflammatory bowel disease (IBD). Antitumor necrosis factor α (anti-TNF) agents are commonly used as the first biologic in clinical practice, and nearly all pivotal studies of induction therapy enrolled patients with and without prior use of anti-TNF therapy. This narrative review presents a reasonable approach to devising treatment sequences, examining the magnitude of benefit for each drug *versus* placebo or active comparator and then considering how that benefit changes with prior anti-TNF treatment. Data from ULTRA 2, GEMINI 1, VARSITY, and True North in patients with ulcerative colitis indicate that induction adalimumab, vedolizumab, and ozanimod showed lower clinical remission rates after anti-TNF therapy, while UNIFI, OCTAVE 1&2, and U-ACHIEVE/U-ACCOMPLISH show ustekinumab, tofacitinib, and upadacitinib did not. In patients with Crohn’s disease, endoscopic remission or mucosal healing after induction therapy rather than clinical remission as well as assessment of persistent endoscopic remission are good measures of long-term disease outcomes. Considering the drugs for which data on endoscopic remission rates are available, EXTEND and GEMINI 2&3 show adalimumab and vedolizumab with persistently lower endoscopic remission rates after prior anti-TNF therapy, while IM-UNIFI, SEAVUE, and FORTIFY show ustekinumab and risankizumab did not. Data from the multicenter retrospective EVOLVE study indicate that the effectiveness of anti-TNF therapy does not seem to be significantly impacted by prior vedolizumab therapy, and may further suggest the benefit of using vedolizumab as a first-line biologic. As adverse event rates remain low across all treatments, the magnitude of harm from untreated or poorly treated disease far outweighs harm from any individual therapy. Regardless of the treatment sequence, careful monitoring for early signs of treatment nonresponse and switching to another potentially highly active therapy are critical to effective management of IBD.

**Figure:** Video Abstract Please click on the image to play the video (also available as supplemental material).

## Introduction

Over the past two decades, 11 biologic agents have been approved for use in most countries for the treatment of moderate-to-severe inflammatory bowel disease (IBD).

The tumor necrosis factor (TNF)-α antagonists infliximab^1,2^ and adalimumab^3,4^ (and their equivalent biosimilars) are used in both ulcerative colitis (UC) and Crohn’s disease (CD), as are the anti-α4β7-integrin vedolizumab^5,6^ and the anti-interleukin (IL)12/23 ustekinumab.^7^ The anti-TNF golimumab,^8^ the JAK inhibitors tofacitinib^9^ and upadacitinib,^10^ and the sphingosine 1-phosphate receptor modulator ozanimod^11^ are used in UC only, while the anti-TNF certolizumab pegol,^12^ the anti-integrin natalizumab,^13^ and the anti-IL23 risankizumab^14,15^ are used in CD only.

After induction therapy with each of these drugs, fewer than 20% of patients show clinical remission, and the rate improves to only half of all patients after treatment with maintenance therapy. This, in turn, leads to low rates of treatment persistence. Approximately one-third of patients switch to a second biologic after a year, and 20% continue on to a third drug.^16^ Whether the consequences of multiple years of ineffectual treatment can be avoided through better treatment sequencing is critical to consider.

Because of the long experience with anti-TNF agents, they are commonly used as the first biologic in clinical practice. However, management guidelines offer conflicting recommendations on whether that is best. For CD patients, the European Crohn’s and Colitis Organisation (ECCO) recommends using any anti-TNF therapy in the first line^17^ while the American College of Gastroenterology recommends any anti-TNF or vedolizumab^18^ and the American Gastroenterological Association (AGA) recommends infliximab, adalimumab, or ustekinumab.^19^ For UC patients, AGA recommends using infliximab or vedolizumab first^20^ but ECCO does not overtly favor any individual treatment or class of treatments over another as first-line therapy.^21^

These conflicting recommendations largely stem from the difficulties associated with comparing treatment effectiveness across trials, particularly as many have different designs. In the past few years, at least five network meta-analyses attempted to correct for these differences by extracting data from randomized controlled trials in IBD, indirectly comparing results, and rank-ordering the drugs according to their likelihood of achieving clinical remission with induction therapy. Yet, here, too, no consensus has emerged. In two analyses in UC, one ranked infliximab and vedolizumab first overall but ranked ustekinumab or tofacitinib first for patients previously treated with anti-TNF therapy,^22^ while the other ranked upadacitinib first with no distinction for prior treatment.^23^ One analysis in CD ranked infliximab or adalimumab first and adalimumab or risankizumab second,^24^ while another ranked infliximab first overall, but ranked risankizumab first when dividing patients into biologic-naïve and biologic-exposed cohorts.^25^ Putting these data together suggests that patients with UC who are naïve to anti-TNF therapy could start with infliximab, vedolizumab, or upadacitinib, and then switch to ustekinumab or tofacitinib after treatment failure, while TNF-naïve patients with CD could start with infliximab or adalimumab, and then switch to risankizumab as second-line therapy.

However, utility of these analyses may be limited. In the UC studies, one of the analyses^22^ considered neither upadacitinib nor ozanimod because they were not yet available, while the other^23^ included the investigational etrolizumab, whose study in IBD has since been discontinued. Furthermore, the CD studies ranked the therapies only according to their ability to achieve clinical remission, yet, as discussed below, endoscopic remission or mucosal healing may be more informative in predicting long-term disease outcomes.^26,27^

This narrative review presents an alternative approach, considering each trial on its own and examining the magnitude of benefit for the drug *versus* placebo or active comparator. Because nearly all pivotal studies of induction therapy enrolled patients with and without prior use of anti-TNF therapy, it is therefore possible to devise treatment sequences based on how that benefit changes with prior anti-TNF treatment.

Although definitions of clinical and endoscopic remission vary somewhat between studies, and one cannot derive statistically significant results from subgroup post hoc comparisons, review of available data can nevertheless offer some insight into whether each agent might best be used first or second (or third) in a treatment sequence in TNF-naïve and TNF-exposed patients. Thus, the sections to follow analyze studies of adalimumab, vedolizumab, ustekinumab, tofacitinib, upadacitinib, ozanimod, and risankizumab in UC and/or CD using this methodology. (Certolizumab pegol, golimumab, and natalizumab are not discussed here as there are fewer data available and they are less commonly used in clinical practice.) First, study data on induction therapy in UC are considered for each biologic based on clinical remission rates before and after anti-TNF therapy. A similar strategy is employed for study data on endoscopic remission rates with maintenance therapy in CD. Next, efficacy of biologic therapy is considered when anti-TNF is *not* used first, and finally, considerations are presented for potential treatment sequencing.

## Clinical remission rates with induction therapy in UC

Broadly speaking, in patients with UC, induction adalimumab, vedolizumab, and ozanimod showed lower clinical remission rates after anti-TNF therapy, while ustekinumab, tofacitinib, and upadacitinib did not (Table 1).

**Table 1.** Clinical remission in UC with and without prior use of anti-TNF therapy.*

	Overall			TNF naive			TNF exposed		
	Drug	Placebo	Difference	Drug	Placebo	Difference	Drug	Placebo	Difference
Drugs that show lower clinical remission rates after anti-TNF therapy									
Adalimumab (ULTRA 2)	16.5%	9.3%	7.2	21.3%	11.0%	10.3	9.2%	6.9%	2.3
Vedolizumab (GEMINI 1)	16.9%	5.4%	11.5	23.1%	6.6%	16.5	9.8%	3.2%	6.5
Ozanimod (True North)	18.4%	6.0%	12.0	22.1%	6.6%	15.5	10%	4.6%	5.4
Drugs that show similar clinical remission rates before and after anti-TNF therapy									
Ustekinumab (UNIFI)	15.5%	5.3%	10.2	18.4%	9.9%	8.5	12.7%	1.2%	11.5
Tofacitinib (OCTAVE 1, OCTAVE 2)	18.5%, 16.6%	8.2%, 3.6%	10.3, 13.0	23.7%, 22.1%	12.5%, 8.5%	11.2, 13.5	12.3%, 12.0%	0.8%, 0.0%	11.5, 12.0
Upadacitinib (U-ACHIEVE, U-ACCOMPLISH)	16.0%, 33.0%	5.0%, 4.0%	21.0, 29.0	35.2%, 37.5%	9.2%, 5.9%	26.0, 31.6	29.6%, 17.9%	2.4%, 0.4%	27.2, 17.5

* Data shown are from independent trials with different trial designs. It is not possible to compare across trials. Difference represents absolute change in percentage points between drug and placebo.

TNF, tumor necrosis factor; UC, ulcerative colitis.

### Drugs that show lower clinical remission rates after anti-TNF therapy

#### Adalimumab

The ULTRA 2 study^3^ compared adalimumab *versus* placebo in patients with moderate-to-severe UC. At week 8, there was a 7.2 percentage-point difference in the rate of clinical remission for the entire cohort (16.5% for adalimumab *versus* 9.3% for placebo). Among those naïve to anti-TNF, the difference was even greater at 10.3 points (21.3% *versus* 11.0%). However, the advantage for adalimumab was lost among the anti-TNF-exposed patients, where a difference of only 2.3 percentage points was noted (9.2% *versus* 6.9%). A similar pattern was seen when comparing clinical response rates. Here, a difference in the rate between drug and placebo was 20.7 points for anti-TNF-naïve (59.3% *versus* 38.6%) but only 8.0 in TNF-exposed patients (36.7% *versus* 28.7). The importance of considering prior anti-TNF use was particularly obvious when assessing mucosal healing rates: in TNF-naïve patients, there was a 14.1-point difference between adalimumab and placebo (49.3% *versus* 35.2%) but only a 1.9-point difference in TNF-exposed patients (28.6% *versus* 26.7%).

#### Vedolizumab

Analysis of data from the GEMINI 1 study^5,28^ also showed lower rates of efficacy in TNF-exposed than in TNF-naïve patients treated with induction vedolizumab *versus* placebo. At week 8, the difference in clinical response rates for the full cohort was 26.1 percentage points between vedolizumab and placebo (47.1% *versus* 25.5%) and a difference of 11.5 points for clinical remission (16.9% *versus* 5.4%). Yet, when prior anti-TNF therapy use was considered, the difference in clinical response rate was greater in the TNF-naive group (difference 26.8; 53.1% *versus* 26.3%) than in the TNF-exposed group (difference 18.4; 39.0% *versus* 20.6%), and was considerably greater when evaluating clinical remission rates in TNF-naïve (difference 16.5; 23.1% *versus* 6.6%) than TNF-exposed (difference 6.5; 9.8% *versus* 3.2%) groups. An even greater difference between TNF-naïve and TNF-exposed patients was seen in mucosal healing rates, with a difference of 24.2 percentage points in the TNF-naïve cohort (49.2% *versus* 25.0%) and only 9.9 points in the TNF-exposed cohort (30.5% *versus* 20.6%).

The phase IIIb VARSITY study comparing adalimumab with vedolizumab in patients with UC is one of the few prospective head-to-head studies in IBD.^29^ Results demonstrated superiority for vedolizumab over adalimumab in achieving clinical remission, with a difference of 8.8 percentage points between the two arms (31.2% *versus* 22.5%). But when evaluating only patients with prior exposure to an anti-TNF therapy other than adalimumab, clinical remission rate with both drugs fell to 20.3% and 16.0%, respectively. Similar outcomes were noted when evaluating the secondary endpoint of endoscopic remission: in the overall population, there was a 12-point difference between vedolizumab and adalimumab (39.7% *versus* 27.7%), but in the TNF-exposed group, rates were lower in both groups (26.6% *versus* 21.0%).

#### Ozanimod

The True North study of induction ozanimod^11^ assessed clinical remission, clinical response, and endoscopic remission at 10 weeks in patients previously treated with an anti-TNF, vedolizumab, or tofacitinib, but subgroup analysis was only done for clinical remission in those with prior anti-TNF exposure. In the overall population, there was a difference of 12.4 percentage points in the clinical remission rate between ozanimod and placebo (18.4% *versus* 6.0%), and the magnitude of benefit was even somewhat better in those who were naïve to anti-TNF therapy (15.5-point difference; 22.1% *versus* 6.6%). However, in those previously treated with anti-TNF therapy, clinical remission rates were considerably worse, with a difference of only 5.4 percentage points (10% *versus* 4.6%) between ozanimod and placebo, which was not statistically significant.

#### Summary

Despite differences in the design of ULTRA 2, GEMINI 1, VARSITY, and True North studies, and despite the fact that each of the drugs studied has a different mechanism of action, the studies all showed that the use of adalimumab, vedolizumab, and ozanimod yield lower rates of clinical remission, clinical response, and/or endoscopic remission in UC patients previously treated with anti-TNF therapy. These data suggest that vedolizumab and ozanimod would optimally be used earlier in the treatment sequence in patients with UC, before initiating anti-TNF therapy, and that in general switching to a second anti-TNF therapy after initial failure is not an optimal strategy.

### Drugs that show similar clinical remission rates before and after anti-TNF therapy

#### Ustekinumab

In the ustekinumab UNIFI induction study in moderate-to-severe UC,^7^ prior treatment did not seem to have an effect on clinical remission rate, although patients were stratified based on prior exposure to any biologic therapy, including an anti-TNF or vedolizumab. At week 8, there was a 10.2 percentage point difference between ustekinumab and placebo in the overall cohort (15.5% *versus* 5.3%), an 8.5-point difference in biologic-naïve patients (18.4% *versus* 9.9%), and an 11.5-point difference in biologic-exposed patients (12.7% *versus* 1.2%). Clinical response rates were also quite similar regardless of prior biologic use. In the overall cohort, there was a 30.5 percentage point difference between ustekinumab and placebo (61.8% *versus* 31.3%), a 30.9-point difference in biologic naïve patients (66.7% *versus* 35.8%), and a 29.9-point difference in biologic-exposed patients (57.2% *versus* 27.3%). The difference between ustekinumab and placebo was similarly minimal when considering the endpoint of endoscopic improvement, with a 12.1-point difference in the biologic-naïve group (33.3% *versus* 21.2%) and a 14.3-point difference (21.1% *versus* 6.8%) in the biologic-exposed group.

#### Tofacitinib

In the OCTAVE 1 and OCTAVE 2 studies of induction tofacitinib,^9^ prior exposure to anti-TNF therapy did not seem to alter outcomes. The difference in clinical remission rates at 8 weeks between tofacitinib and placebo in the overall group was 10.3 percentage points in OCTAVE 1 (18.5% *versus* 8.2%) and 13 percentage points in OCTAVE 2 (16.6% *versus* 3.6%). On subgroup analysis, TNF-naïve and TNF-exposed patients also showed similar outcomes. In OCTAVE 1, the difference between tofacitinib and placebo in the TNF-naïve group was 9.4 points (25.2% *versus* 15.8%) and in the TNF-exposed it was 11.1 points (12.6% *versus* 1.5%), while in OCTAVE 2, TNF-naïve patients saw a 13.5-point difference (22.1% *versus* 8.5%), and TNF-exposed patients saw a 12-point difference (12.0% *versus* 0%). Mucosal healing rates were also unaffected by prior anti-TNF use. In OCTAVE 1, there was a difference of 15.7 percentage points (31.3% *versus* 15.6%) in the overall population and 17.9 points (24% *versus* 6.2%) in TNF-exposed patients, while in OCTAVE 2 there was a difference of 16.8 points in the overall population (28.4% *versus* 11.6%) and 15.6 points (21.8% *versus* 6.2%) in TNF-exposed patients.

#### Upadacitinib

Induction upadacitinib *versus* placebo was evaluated in a pair of studies^10^ that showed largely similar results somewhat different magnitudes of benefit in biologic-naïve and biologic-exposed patients, but the difference was fairly small and both groups in each study showed clinical remission rates similar to that in the overall cohort. The clinical remission rate at week 8 in the overall population in U-ACHIEVE was 26% with upadicitinib *versus* 5% with placebo, or a difference of 21 percentage points, and was 33% *versus* 4%, or a difference of 29 points, in U-ACCOMPLISH. Among those with no prior biologic use (i.e. anti-TNF, vedolizumab, or ustekinumab), the difference in clinical remission rates was similar to the overall population in both U-ACHIEVE (26-point difference; 35.2% *versus* 9.2%) and U-ACCOMPLISH (31.6-point difference; 37.5% *versus* 5.9%). Biologic-exposed patients in U-ACCOMPLISH (27.2-point difference; 29.6% *versus* 2.4%) and in U-ACHIEVE showed generally similar outcomes (17.5-point difference; 17.9% *versus* 0.4%). Of note, differences in endoscopic remission were more noticeable, with a difference of 13 percentage points (14% *versus* 1%) in the overall population in U-ACHIEVE and a similar magnitude of benefit of 16 percentage points (18% *versus* 2%) in U-ACCOMPLISH. This difference was unchanged or improved when restricting to only the biologic-naïve cohort [16.5 points (19.1% *versus* 2.6%) and 21.4 points (23.8% *versus* 2.4%), respectively], and fell when restricting to the biologic-exposed cohort [8.9 points (8.9% *versus* 0%) and 11.5 points (12.7% *versus* 1.2%), respectively].

#### Summary

Data from UNIFI, OCTAVE 1&2, and U-ACHIEVE/U-ACCOMPLISH show a similar magnitude of benefit in clinical remission rates when induction ustekinumab, tofacitinib, and upadicitinib are compared with placebo regardless of treatment history, suggesting that saving these agents for a later line of therapy might be a prudent approach.

## Endoscopic remission rates with maintenance therapy in CD

As indicated above, in patients with CD, endoscopic remission or mucosal healing after induction therapy rather than clinical remission is likely a better predictor of long-term disease outcomes, and therefore a better measure to use when considering the magnitude of treatment benefit. Meta-analysis of 10 studies^27^ showed that upon assessment ⩾50 weeks after study onset, patients with mucosal healing at first endoscopic assessment were more likely to show significantly higher rates of clinical remission [68.9% *versus* 42.5%; odds ratio (OR): 2.80 (95% confidence interval (CI), 1.91–4.10) and mucosal healing (93.5% *versus* 18%; OR: 14.30 (95% CI, 5.57–36.74)], as well as a trend toward lower rates of freedom from CD-related surgery [93% *versus* 831.1; OR: 2.22 (95% CI, 0.86–5.69)].

Importantly, the benefit from endoscopic remission persists. In long-term follow-up of the CALM study,^26^ patients who showed any measure of mucosal healing at 48 weeks at study end showed decreased risk of disease progression after a median of 3 years, but risk reduction was greatest in those who achieved deep remission, defined as Crohn’s Disease Activity Index < 150, Crohn’s Disease Endoscopic Index of Severity < 4 with no deep ulcerations, and no steroids for ⩾8 weeks, with an adjusted HR of 0.19 (95% CI, 0.07–0.31) compared with those not in remission. These data suggest that assessment of persistent endoscopic remission is a good measure of long-term disease outcomes.

Not all studies of biologic therapy in CD include endoscopic remission or mucosal healing as an endpoint, potentially limiting its use in defining optimal treatment sequences for patients with CD. Considering the drugs for which data on endoscopic remission rates are available, broadly speaking, only adalimumab and vedolizumab showed persistently lower endoscopic remission rates after prior anti-TNF therapy, while ustekinumab and risankizumab did not (Table 2).

**Table 2.** Endoscopic remission in CD with and without prior use of anti-TNF therapy.*

	Overall			TNF naive			TNF exposed		
	Drug	Placebo	Difference	Drug	Placebo	Difference	Drug	Placebo	Difference
Drugs that show lower endoscopic remission rates after anti-TNF therapy									
Vedolizumab (VERSIFY)^a^									
26 weeks	11.9%	–	–	19.6%	–	–	5.5%	–	–
52 weeks	17.9%			25.0%			8.3%		
Adalimumab (EXTEND)^b^									
12 weeks	27.0%	13.0.0%	14.0	31.3%	11.5%	9.0	31.3%	11.5%	19.8
52 weeks	24.0%	0%	24.0	25.0%	0%	25.0	23.3%	0%	23.3
Drugs that show similar endoscopic remission rates before and after anti-TNF therapy									
Ustekinumab (IM-UNITI)^c^									
12 weeks	47.7%	29.9%	17.8	43.9%	17.1%	26.8			
44 weeks	37.0%	25.0%	12.0	–	–	–			
Risankizumab (FORTIFY)^d^									
180 mg dose	47.1%			63.6%			40.7%		
360 mg dose	46.8%	22.0%	~25	53.8%	26.8%	~32	44.1%	20.3%	~22

* Data shown are from independent trials with different trial designs. It is not possible to compare across trials. Difference represents absolute change in percentage points between drug and placebo.

a Assessed for endoscopic remission defined as SES-CD ⩽ 4.

b Assessed for mucosal healing rate defined as the absence of mucosal ulceration.

c Assessed for clinically meaningful endoscopic improvement rate defined as SES-CD ⩾ 3 points from baseline.

d Assessed at 52 weeks for endoscopic response defined as a decrease in SES-CD ⩾ 50% from baseline.

CD, Crohn’s disease; SES-CD, Simplified Endoscopic Severity Crohn’s disease; TNF, tumor necrosis factor.

### Drugs that show lower endoscopic remission rates after anti-TNF therapy

#### Adalimumab

The EXTEND study^30^ evaluated mucosal healing rates defined as the absence of mucosal ulceration in patients started on 160 mg adalimumab at week 0, switched to 80 mg at week 2, then randomized at week 4 to 40 mg or placebo. At week 12, there was a difference of 14 percentage points (27% *versus* 13%) in the mucosal healing rate between those treated with continuous adalimumab *versus* those who switched to placebo. The magnitude of benefit for continuous adalimumab was maintained in the TNF-exposed population (23.3% *versus* 14.3%) but was considerably greater in the TNF-naïve population (31.3% *versus* 11.5%).

#### Vedolizumab

In the pivotal GEMINI 2 and GEMINI 3 studies of vedolizumab, patients with CD were assessed for clinical remission after 6 weeks or 10 weeks of induction therapy, respectively.^6,31,32^ In the phase IIIb VERSIFY study,^33^ patients treated with vedolizumab were assessed for endoscopic remission, defined as Simplified Endoscopic Severity Crohn’s disease (SES-CD) score ⩽4, at 26 weeks and again 52 weeks. In the overall cohort, endoscopic remission was seen in 11.9% at 26 weeks and in 17.9% at 52 weeks. However, consistent with activity of vedolizumab in TNF-exposed patients with UC, patients with CD treated with vedolizumab after anti-TNF therapy showed considerably worse outcomes. At 26 weeks, 19.6% of TNF-naïve patients achieved endoscopic remission compared with only 5.5% of TNF-exposed patients, and a similar discrepancy of 25.0% *versus* 8.3% was seen at 52 weeks.

These data clearly indicate that reserving vedolizumab for CD patients naïve to anti-TNF therapy is the best option for maximizing benefit from this agent, and that switching to a second anti-TNF therapy after first-line failure is not an optimal approach.

### Drugs that show similar endoscopic remission rates before and after anti-TNF therapy

#### Ustekinumab

Ustekinumab was studied in the UNITI-1 study of TNF-exposed patients as well as in the UNITI-2 study of patients who were TNF-naïve or did not meet criteria for having failed prior TNF; those who completed induction were enrolled in the IM-UNITI maintenance ustekinumab study.^34^ Patients enrolled in the endoscopy substudy underwent assessment for SES-CD score at week 0 of induction, at week 8 of induction, which also served as week 0 of maintenance, and at week 44 of maintenance.^35^ There was a difference of 17.8 percentage points in endoscopic improvement (SES-CD ⩾3 points from baseline) in the overall population at week 8 (47.7% *versus* 29.9%) and a 12-point difference at week 44 (37% *versus* 25%). Rates with maintenance therapy at week 44 stratified by prior treatment use were not described in IM-UNITI. Results of the phase IIIb SEAVUE study^36^ comparing adalimumab and ustekinumab do not suggest a particular advantage of ustekinumab to achieve the desired outcome of endoscopic remission after 1 year of treatment. At 52 weeks, rates of endoscopic remission, defined as SES-CD ⩽3, were largely equivalent (31% with adalimumab *versus* 29% with ustekinumab) regardless of baseline SES-CD score.

#### Risankizumab

The ADVANCE study of induction risankizumab enrolling both biologic-naïve and biologic-exposed patients, as well as MOTIVATE enrolling only biologic-exposed patients assessed clinical remission rates and endoscopic response (50% reduction from baseline SES-CD) at 12 weeks.^14^ The phase III FORTIFY study of maintenance risankizumab (180 mg or 360 mg)^15^ enrolled patients from ADVANCE and MOTIVATE who had achieved clinical response at week 12 or 24, and assessed them at 52 weeks for both clinical remission and endoscopic response defined as a decrease in SES-CD ⩾50% from baseline. In the overall cohort, there was a difference of approximately 25 percentage points in endoscopic response rates at 52 weeks between the two doses of risankizumab *versus* placebo (47.1% and 46.8%, respectively, *versus* 22.0%). Among biologic-naïve patients, the magnitude of benefit was higher, with a difference of approximately 32 percentage points between the risankizumab doses and placebo (63.6% and 53.8%, respectively, *versus* 26.8%), but those with prior biologic failure showed an approximately 22-point magnitude of benefit (40.7% and 44.1%, respectively, *versus* 20.3%), similar to that of the overall population.

#### Summary

Although IM-UNIFI, SEAVUE, and FORTIFY differed slightly in their definitions of endoscopic endpoints, they all showed that maintenance therapy with ustekinumab and risankizumab can yield reasonable rates of persistent endoscopic remission and/or mucosal healing regardless of anti-TNF treatment history. This suggests that both of these drugs could be used as first-line therapy in both TNF-naïve and TNF-exposed patients. However, as only FORTIFY included endoscopic response as a co primary endpoint, risankizumab has the highest quality data examining this endpoint. Furthermore, considering the data from VERSIFY showing lower rates with vedolizumab after anti-TNF therapy, and the limited controlled data for ustekinumab after anti-TNF therapy, at this time neither of these medications should be first choice in anti-TNF-exposed patients.

## Efficacy of treatment when anti-TNF is *not* used first

Because the anti-TNF agents were developed first and are commonly used first in clinical practice, there are no prospective studies of anti-TNF therapies in patients previously treated with other biologics. Nevertheless, retrospective data can be highly useful in assessing outcomes in this patient population, particularly when considered alongside prospective data.

EVOLVE was a multicenter retrospective study of 1095 patients with IBD, including 604 with UC and 491 with CD, who were treated with vedolizumab or anti-TNF as a first biologic therapy and followed over 24 months^37^ (Table 3). Rates of clinical remission, clinical response, and mucosal healing were high with both agents, and were similar between the groups within each disease. Treatment persistence was longer in UC patients treated with first-line vedolizumab, which may in part have been due to the lower rates of both serious infection and serious adverse events with vedolizumab. Most important, because some patients switched to anti-TNF therapy after vedolizumab, it was possible to compare clinical effectiveness of first-line anti-TNF *versus* second-line anti-TNF after first-line vedolizumab. Although the cohorts were too small to make definitive conclusions, clinical remission was similar at both 3 months and 6 months in UC patients using anti-TNF in the first line and in the second line [9.7% *versus* 11.0% (*p* = 0.92) and 19.6% *versus* 14.7% (*p* = 0.69), respectively] but rates were higher in CD patients using anti-TNF in the second line at these same timepoints [22.9% *versus* 49.2 (*p* = 0.02) and 36.2% *versus* 74.6% (*p* < 0.01], respectively), but the number of patients in the second line group was very small.

**Table 3.** Clinical effectiveness in EVOLVE.

	UC			CD		
First-line vedolizumab or first-line anti-TNF over 24 months						
	First-line vedolizumab	First-line anti-TNF	*p* Value	First-line vedolizumab	First-line anti-TNF	*p* Value
Clinical remission	65.9%	48.6%	0.09	76.6%	68.5%	0.10
Clinical response	88.3%	86.2%	0.64	84.0%	72.1%	0.27
Mucosal healing	86.6%	80.6%	0.66	100%	90.4%	0.12
First-line anti-TNF or second-line anti-TNF at 3 or 6 months						
	First-line anti-TNF	Second-line anti-TNF	*p* Value	First-line anti-TNF	Second-line anti-TNF	*p* Value
Clinical remission at 3 months	9.7%	11.0%	0.92	22.9%	49.2%	***0.02***
Clinical response at 3 months	38.4%	44.8%	0.54	30.1%	41.3%	0.52
Clinical remission at 6 months	19.6%	14.7%	0.69	36.2%	74.6%	***<0.01***
Clinical response at 6 months	57.1%	61.1%	0.58	43.5%	74.8%	0.13

*p* Values in bold italics are statistically significant.

CD, Crohn’s disease; TNF, tumor necrosis factor; UC, ulcerative colitis.

Thus, if data from VARSITY, the GEMINI studies, and VERSIFY indicate that maximal effect for vedolizumab is seen when it is used before anti-TNF therapy, data from EVOLVE further indicate that effectiveness of anti-TNF therapy does not seem to be significantly impacted by prior vedolizumab therapy, and may further suggest the benefit of using vedolizumab as a first-line biologic.

## Considerations for potential treatment sequencing

Weighing all of the data presented above, when considering clinical remission rates in UC, anti-TNF therapy is likely not the best choice for first-line therapy in biologic-naïve patients. Most non-TNF biologic therapies show worse results when used after anti-TNF, while the reverse does not necessarily seem be true. Thus, a sequence of a biologic other than anti-TNF followed by an anti-TNF is likely to allow for maximal benefit from both agents (Table 4).

**Table 4.** Potential sequence of biologic agents.

	UC (considering magnitude of benefit for clinical remission)		CD (considering magnitude of benefit for endoscopic remission/mucosal healing)	
	Anti-TNF naïve	Anti-TNF exposed	Anti-TNF naïve	Anti-TNF exposed
First line*	Vedolizumab, ozanimod, or ustekinumab	Ustekinumab, tofacitinib, or upadacitinib	Risankizumab, ustekinumab, vedolizumab	Risankizumab or ustekinumab
Second line*	Tofacitinib or upadacitinib			
Third line	Anti-TNF			

* No sequence is recommended within each category.

CD, Crohn’s disease; TNF, tumor necrosis factor; UC, ulcerative colitis.

For patients with CD, the priority should be placed on a sequence of therapies maximizing the likelihood that a patient will achieve endoscopic remission. The most recent data emphasizing the value of this target is from CALM showing markedly improved long-term clinical outcomes with endoscopic remission.^26^ Risankizumab, ustekinumab, vedolizumab, and adalimumab all induce some measure of endoscopic response or mucosal healing, and only vedolizumab shows poorer outcomes after an anti-TNF therapy. Thus, a sequence of a biologic other than an anti-TNF followed by an anti-TNF is attractive for TNF-naïve patients, while only risnakizumab or ustekinumab would be recommended in TNF-exposed patients (Table 4).

There may also be a mechanistic reason that ustekinumab and risankizumab would specifically benefit patients previously treated with an anti-TNF therapy.^38^ Nonresponders to anti-TNF therapy show an increase in apoptosis-resistant, IL23-positive T cells, which promote inflammation. In this setting, an IL23 inhibitor could be particularly attractive. Interestingly, studies comparing ustekinumab with vedolizumab in patients with CD who failed prior anti-TNF therapy would seem to support this.^39,40^ One found a difference of 19.1 percentage points (35.6% *versus* 16.5%) in clinical remission rate at 8 weeks and a 16.3-point difference (42.2% *versus* 25.9%) at 52 weeks in favor of ustekinumab,^39^ while the other found a similar 16.1-point difference (54.4% *versus* 38.3%) at week 48 in favor of ustekinumab.

Another consideration for treatment sequencing is safety. Network meta-analyses evaluating treatment efficacy show largely similar rates of adverse events across all drugs,^23,24^ data from EVOLVE^37^ demonstrated a lower rate of serious infections in biologic-naïve patients started on vedolizumab compared to anti-TNF, and ustekinumab in CD^41^ showed no new safety signals emerging over time. There are some suggestions that ustekinumab may be associated with a lower rate of serious infection in patients with CD compared with both anti-TNF and vedolizumab, and, conversely, that vedolizumab may be associated with a lower risk of serious infection in UC compared with anti-TNF therapy.^42^ Nevertheless, as rates remain low across all treatments, safety is not likely to be a main driver of treatment choice. Indeed, as the magnitude of harm from untreated or poorly treated disease far outweighs harm from any individual therapy, the primary focus should be on maximizing benefit from each available biologic agent over time.

Finally, and perhaps most important, regardless of study data, it is critical that every treatment choice be individualized to the patient. We cannot yet quantify the risk of disease progression and disease complications for any IBD phenotype, and risk factors for rare side effects of biologic therapy must always be considered. The potential of a single biologic agent to treat both IBD and a concomitant immune mediated disorder might also drive selection. For example, risankizumab or ustekinumab might be a good option for a patient with both CD and psoriasis, while an anti-TNF might be optimally selected for a patient with IBD and concomitant uveitis. Availability of drug, cost, and patient preference for schedule/delivery are also likely to affect treatment choice. Ultimately, regardless of the treatment sequence selected, careful monitoring for early signs of treatment nonresponse and a recognition that nonresponse to one therapy still leaves the door open to other therapies are key to effective management of IBD.

## Declarations

**Ethics approval and consent to participate:** Not applicable.

**Consent for publication:** Not applicable.

**Availability of data and materials:** Not applicable.
